# Supplementary material for: Characterisation of Orthohantavirus Serotypes in Human Infections in Kazakhstan
Source: Viruses. 2025 Jun 28;17(7):925. doi: 10.3390/v17070925 (PMC12298176; doi:10.3390/v17070925)
Supplement: Supplementary file 1 [file viruses-17-00925-s001.zip › viruses-3663321-supplementary.pdf]

Supplementary Table S1. Seropositivity of IgM and IgG antibodies against orthohantavirus by ELISA in patients with suspected cases of HFRS in West Kazakhstan region and Almaty city in 2018 and 2019.

Sampling region	ELISA negative (%)	ELISA IgM positive only (%)	ELISA IgG positive only (%)	ELISA IgM and IgG positive
**Almaty city (n=82)**	70 (85.4)	2 (2.4)	10 (12.2)	0
**West Kazakhstan region (n=57)**	28 (49.1)	0	24 (42.1)	5 (8.8)
**Total (n=139)**	98 (5.0)	2 (24.5)	34 (70.5)	5

Supplementary Table S2. Clinical signs of tested subjects

**Symptoms**	**IgM negative [%] (n=132)**	**IgM positive [%] (n=7)**
Fever	98	100
Headache	89	57
Weakness	65	71
Arthralgia	30	14
Back pain	15	43
Stomach pain	61	29
Cough	8	14
Sore throat	13	14
Visual disturbance	8	14
Enlarged lymph nodes	9	0
Signs of haemorrhage	13	0
Thrombocytopenia (PLT < 180 g/L, reference 180-320 g/L)	23	100

Supplementary Table S3. Characteristics of Orthohantavirus IgG seropositivity in patients with suspected HFRS in West Kazakhstan region and Almaty city (2018-2019).

	Orthohantavirus ELISA seronegative IgG 1st and/or 2nd serum IgG (n=105)	Orthohantavirus ELISA seropositive IgG 1st and/or 2nd serum (n=34)	Total (n=139)	p value
**Region**				< 0.001[1]
West Kazakhstan	33 (57.9%)	24 (42.1%)	57	
Almaty city	72 (87.8%)	10 (12.2%)	82	
**Living area**				0.004[1]
Rural area	23 (57.5%)	17 (42.5%)	40	
Urban area	82 (82.8%)	17 (17.2%)	99	
**Gender**				0.841[1]
Male	61 (74.4%)	21 (25.6%)	82	
Female	44 (77.2%)	13 (22.8%)	57	
**Hospital Type**				0.086[1]
Infectious Disease Hospital	69 (71.1%)	28 (28.9%)	97	
Nephrology Department	36 (85.7%)	6 (14.3%)	42	
**Age**				0.191[3]
Mean (SD)	36.1 (15.0)	39.6 (15.6)	37.0 (15.2)	
Median (Q1, Q3)	31.0 (26.0, 42.0)	37.000 (27.2, 48.7)	32.0 (26.0, 45.0)	
Min - Max	18 - 83	18 - 76	18 - 83	
**Recent geographical movement**				0.522[1]
No	74 (77.1%)	22 (22.9%)	96	
Yes	30 (71.4%)	12 (28.6%)	42	
Missing	1	0	1	
**Nature trip**				0.233[1]
No	85 (78%)	24 (22%)	109	
Yes	20 (66.7%)	10 (33.3%)	30	
Missing	0	0	0	
**Small mammal bite**				1.000[1]
No	104 (75.4%)	34 (24.6%)	138	

	Orthohantavirus ELISA seronegative IgG 1st and/or 2nd serum IgG (n=105)	Orthohantavirus ELISA seropositive IgG 1st and/or 2nd serum (n=34)	Total (n=139)	p value
Yes	1 (100%)	0 (0.0%)	1	
**House type**				0.010[1]
Apartment	62 (84.9%)	11 (15.1%)	73	
House	43 (65.2%)	23 (34.8%)	66	
**Patient reported rodent sightings**				0.843[1]
Never	46 (74.2%)	16 (25.8%)	62	
Yes	59 (76.6%)	18 (23.4%)	77	
**Patient trapped/killed rodents**				0.357[1]
No	91 (74%)	32 (26%)	123	
Yes	14 (87.5%)	2 (12.5%)	16	
**Contact to rodent excreta**				0.431[1]
No	104 (75.9%)	33 (24.1%)	137	
Yes	1 (50%)	1 (50%)	2	
**Vegetation in living area**				0.018[4]
Dense Plantations	66 (84.6%)	12 (15.4%)	78	
Large grass fields	14 (70%)	6 (30%)	20	
Agricultural fields	12 (63.2%)	7 (36.8%)	19	
Other	11 (55%)	9 (45%)	20	
Missing	2	0	2	

[1]Fisher's Exact Test for Count Data, [2]Pearson's Chi^2 test with simulated p-value (based on 5000 replicates), [3]Mann Whitney U test, [4]Fisher's Exact Test for Count Data with simulated p-value (based on 5000 replicates)
